# New distributional and bioacoustic data about *Cicadivetta goumenissa* from Peloponnese, Greece (Hemiptera, Cicadidae)

**DOI:** 10.3897/zookeys.319.4452

**Published:** 2013-07-30

**Authors:** Matija Gogala, Sakis Drosopoulos, Tomi Trilar

**Affiliations:** 1 Slovenian Academy of Sciences and Arts, Novi trg 3, SI-1000 Ljubljana, Slovenia; 2 Elatis 6, GR-14564, Nea Kifisia, Greece; 3 Slovenian Museum of Natural History, Prešernova 20, SI-1000 Ljubljana, Slovenia

**Keywords:** Hemiptera, Cicadidae, *Cicadivetta goumenissa*, geographical distribution, song pattern

## Abstract

*Cicadivetta goumenissa*, a small singing cicada described recently by Gogala et al. (2012), has been previously found only around the village of Goumenissa in northern Peloponnese. We visited this area again in June 2012 and tried to determine the distribution range of this species. We found *Cicadivetta goumenissa* in some further localities, but all within a very small area of about 15 by 25 km. We also made more than one hour of new song recordings and extended our knowledge of the song repertoire of this species.

## Introduction

We found and described this year the new cicada species *Cicadivetta goumenissa* Gogala et al., 2012 in just a small area in northern Peloponnese, Greece (Gogala et al. 2012). As mentioned in the paper, we first discovered this species in 2005 near the village of Goumenissa by its characteristic high pitched song with very fast repetitions of short echemes, interrupted by single long echemes. Until this year, we have never heard such sound pattern or ever collected this cicada in any other parts of the Peloponnese or elsewhere during our field work. Therefore, we decided to visit this area again this year (2012) to get more data on this peculiar cicada and to find out whether *Cicadivetta goumenissa* is really restricted just to this locality and possibly why.

## Materials and methods

We made a field trip to the Peloponnese from 9 to 11 of June, 2012. From previous visits to this area in 2005, 2006 and 2010 we knew that *Cicadivetta goumenissa* could not be found in July or later in the year.

We were searching for the presence of this cicada with bioacoustic equipment described below in localities around Kalavrita, Goumenissa, Pteri, Manesi, Vlasia, Dendra, Mirali, Priolithos, Lagovouni and on Mt. Erymanthos (see Table 1).

For the names and spelling of localities we follow the local inscriptions and transliterations used in the maps of the “Road Editions”, Nr. 4 and 5, Athens (1996).

The distribution map was created by GPS Visualizer (Schneider 2003–2012).

For acoustic detection and recording of songs we used the ultrasonic detector Pettersson D-200 (heterodyne system) with electret microphones of the same producer (frequency range 10–120 kHz), mounted in front of a Telinga reflector (57 cm diameter) or a Renault R-4 front light reflector and connected to the solid state recorders Marantz PMD-660 (sampling rate up to 48 kS) or Zoom H2 (sampling rate up to 96 kS). Due to the high frequency range (see Gogala et al. 2012), we would not have been able to hear the acoustic signals of this cicada species without ultrasonic detectors. For sound analyses we used RAVEN 1.4 (Cornell Lab of Ornithology) and AMADEUS Pro 2.0 (HairerSoft). We first localized cicadas acoustically and then, if possible, collected them with an entomological net.

For the nomenclature of cicadas we are following the publication of Puissant and Sueur (2010).

Collected specimens are deposited in the collection of the Slovenian Museum of Natural History (PMSL). Song recordings are deposited in the Slovenian Wildlife Sound Archive of the same museum. A sample of a selected recording is available on the web pages Songs of European singing cicadas (http://www.cicadasong.eu).

## Results

### Distribution area:

We visited 13 localities in June 2012 and two more in previous years in the neighbourhood of Goumenissa (covering more than 200 km^2^) and tried to detect the characteristic sounds of *Cicadivetta goumenissa*. Reference to other cicada species found is also given (Table 1). Moreover, the visited localities are also shown on the map, where the places with presence of *Cicadivetta goumenissa* are shown in red and those without this species in green (Fig. 2).

During the last eight years we have searched for cicadas in many other localities in Greece, including several field trips to other parts of the Peloponnese (Mt. Menalo, Mt. Parnon, Kastanitsa, Agios Andreas etc.) and have never heard, recorded or collected *Cicadivetta goumenissa*. Therefore we assume that the distribution area of this species is really restricted to a very small area, not exceeding about 15 by 25 km.

**Table 1.** List of localities, where we searched for *Cicadivetta goumenissa*. In the table are given the names of the localities (column 1), dates of visits (column 2), geographic coordinates with elevations (column 3) and names of the cicadas, heard (H), recorded (R), collected (C), observed (O) or photographed (P) in a particular locality (column 4). Included are also data from previous years. The shortened scientific names of species represent the following taxa:<br/> ***C. goumenissa*** – *Cicadivetta goumenissa* Gogala, Drosopoulos & Trilar, 2012<br/> *C. flaveola* – *Cicadivetta flaveola* (Brullé, 1832) <br/> *C. hannekeae* – *Cicadetta hannekeae* Gogala, Drosopoulos & Trilar, 2008<br/> *C. atra* – *Cicadatra atra* Fieber, 1776<br/> *C. orni* – *Cicada orni* Linné, 1758<br/> *D. dimissa* – *Dimissalna dimissa* (Hagen, 1856) <br/> *L. plebejus* – *Lyristes plebejus* (Scopoli, 1763) <br/> *P. annulata* – *Pagiphora annulata* (Brullé, 1832) <br/> *T. haematodes* – *Tibicina haematodes* (Scopoli, 1763) <br/> *T. pygmea* – *Tettigetula pygmea* (Olivier, 1790)

Kalavryta, Goumenissa	9. 6. 2005	38°3.63'N, 22°1.87'E<br/> 780 m	***C. goumenissa*** – R
Kalavryta, Goumenissa,<br/> deviation to Pteri	9. 6. 2005	38°3.10N, 22°1.72E<br/> 740 m	***C. goumenissa*** – C, R<br/> *T. pygmea* – R
	9. 6. 2012	38°3.10'N, 22°1.63'E<br/> 720 m	***C. goumenissa*** – C, R<br/> *T. pygmea* – R<br/> *D. dimissa* – R<br/> *T. haematodes* – H, O
	28. 6. 2006	38°3.10'N, 22°1.72'E<br/> 720 m	***C. goumenissa*** – C, R<br/> *C. atra* – H<br/> *T. haematodes* – C<br/> *D. dimissa* – H<br/> *T. pygmea* – R<br/> *C. flaveola* – C, F<br/> *L. plebejus* – H
	16. 7. 2010	38°2.80'N, 22°1.30'E<br/> 720 m	*C. orni* – H<br/> *L. plebejus* – H<br/> *T. haematodes* – H<br/> *C. flaveola* – C, R, P<br/> *C. atra* – H<br/> *T. pygmea* – R
Kalavryta, Petsaki, deviation to Valta	28. 6. 2006	38°7.07'N, 22°3.32'E<br/> 970 m	*C. flaveola* – R<br/> *C. atra* – H<br/> *D. dimissa* – H<br/> *L. plebejus* – P
Kalavryta, Skepasto (near the road to Goumenissa)	9. 6. 2012	38°1.70'N, 22°2.77'E<br/> 800 m	***C. goumenissa*** – R
Kalavryta,<br/> Mega Spileo	9. 6. 2012	38°5.29'N, 22°10.36'E<br/> 830 m	*D. dimissa* – R
Kalavryta, (deviation to Kerpini and Rogi)	9. 6. 2012	38°3.28'N, 22°8.51'E<br/> 740 m	***C. goumenissa*** – R<br/> *T. pygmea* – R<br/> *T. haematodes* – H<br/> *C. flaveola* – C, R
Kalavryta,<br/> road to Mt. Helmos	10. 6. 2012	38°1.51'N, 22°7.75'E<br/> 970 m	*C. hannekeae* – R<br/> *T. haematodes* – H<br/> *C. flaveola* – R<br/> *D. dimissa* – R
Kalavryta, Manesi	10. 6. 2012	38°0.83'N, 21°56.69'E<br/> 860 m	***C. goumenissa*** – R<br/> *T. pygmea* – R<br/> *T. haematodes* – H<br/> *C. flaveola* – R
Ano Vlasia,<br/> Mt. Erymanthos	10. 6. 2012	37°58.00'N, 21°53.85'E<br/> 1140 m	*C. hannekeae* – R<br/> *T. haematodes* – R
Kalavryta, Lagovouni	10. 6. 2012	37°57.75'N, 22°3.40'E<br/> 770 m	***C. goumenissa*** – R
Kalavryta, Lagovouni, deviation to Kandalos	10. 6. 2012	37°57.11'N, 22°2.92'E<br/> 750 m	*D. dimissa* – R<br/> *T. pygmea* – R<br/> *C. flaveola* – R<br/> *T. haematodes* – R
Klitoria, Priolithos, near the monument	10. 6. 2012	37°54.97'N, 22°2.99'E<br/> 1020 m	*T. pygmea* – R<br/> *C. flaveola* – R
Kalavryta, Kampigadi, Dendra	11. 6. 2012	38°2.40'N, 21°52.94'E<br/> 670 m	***C. goumenissa*** – C, R<br/> *P. annulata* – H<br/> *T. haematodes* – R<br/> *T. pygmea* – R<br/> *C. flaveola* – R<br/> *D. dimissa* – R
Halandritsa,<br/> deviation to Mirali	11. 6. 2012	38°5.75'N, 21°48.76'E<br/> 470 m	*P. annulata* – H<br/> *T. haematodes* – R<br/> *T. pygmea* – R<br/> *D. dimissa* – R
Pteri	28. 6. 2006	38°8.79'N, 22°4.53'E<br/> 1060 m	*C. hannekeae* – R<br/> *C. flaveola* – C, R, P

### Song pattern:

During our last excursion to the Kalavrita and Goumenissa region we made many new recordings (more than one hour) of the *Cicadivetta goumenissa* song. Therefore, we can further comment on our description of the song pattern published previously (Gogala et al. 2012).

The main addition to this description is that the part of the song with longer echemes and a few or even without short echemes interspersed is apparently a regular part of the song and not an exception. Such phrases appear usually in the middle part of a song sequence (Fig. 3). The long echemes (LE) in such phrases can in extreme cases fuse to the longest echemes (XLE), which can last up to 15 s. Therefore the part of a song with such extremely long echemes resembles the so called continuous song of *Cicadatra atra* or related species.

The whole song starts usually, but not always, with a long echeme followed by a sequence of short echemes (SE). The first long echeme may have also a preceding SE. The main part of the song comprises phrases of some SE and one LE, as described previously (Gogala et al. 2012). At the end of a song there are usually longer sequences of SE, interrupted only by a few LE (Fig. 4). The repetition rate of SE is as described previously 19±3 SE/s and at the end of a song usually falls down to 10±2 SE/s. The whole song lasts between a few ten seconds and 3 – 4 minutes, and may be repeated without a longer pause or after a short silent period. We observed many times that the males in this silent period or at the end of a song fly away and change the singing post.

**Figure 1.**
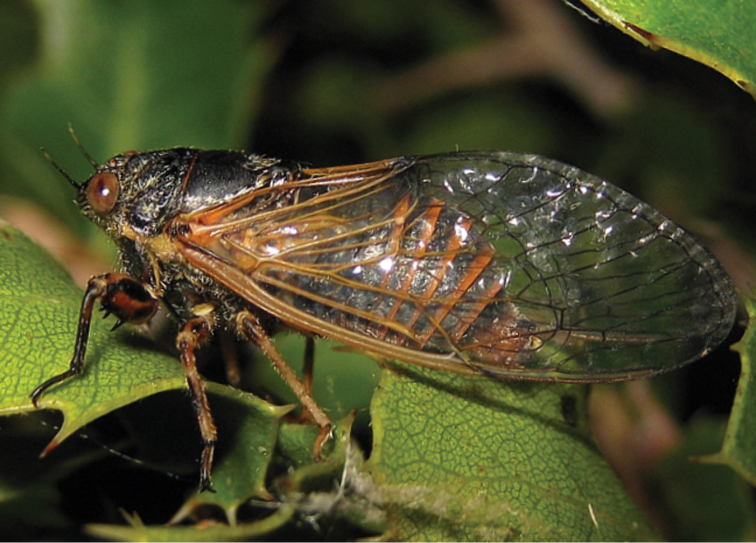
Male of *Cicadivetta goumenissa* on leaves of Kermes Oak (*Quercus coccifera*) (Photo T. Trilar).

In most cases *Cicadivetta goumenissa* males are perching and singing on branches of Kermes Oak (*Quercus coccifera*) as described earlier (Gogala et al. 2012) (Fig. 1). However, in one locality we observed and recorded some males for hours also on one and the same tree of Oriental Plane (*Platanus orientalis*).

**Figure 2.**
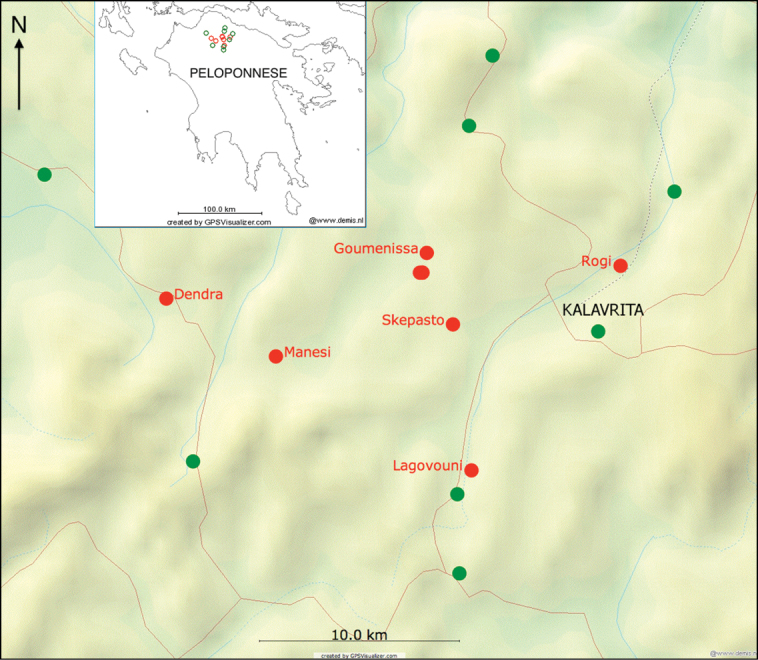
Distribution map of *Cicadivetta goumenissa*. Red points – localities with *Cicadivetta goumenissa*, green points – localities where we searched for but did not find this species. Map created by GPS Visualizer (Schneider 2003-2012). Inset: position of localities on Peloponnese peninsula.

**Figure 3.**
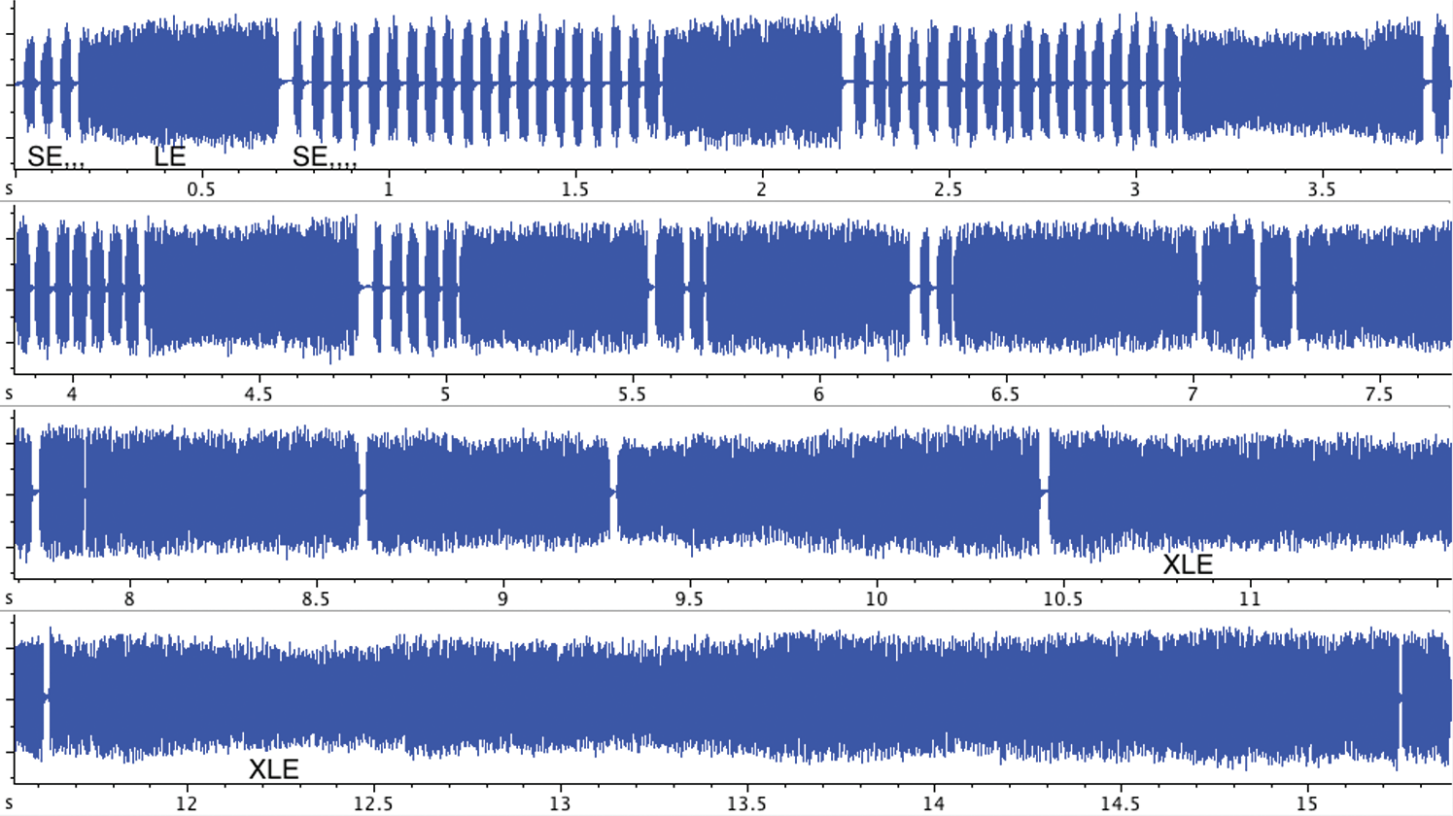
Oscillogram of the song selection of *Cicadivetta goumenissa* with the transition from prevailing phrases with SE sequence followed by LE toward the phrases with very long echemes XLE with a few SE or without them.

**Figure 4.**
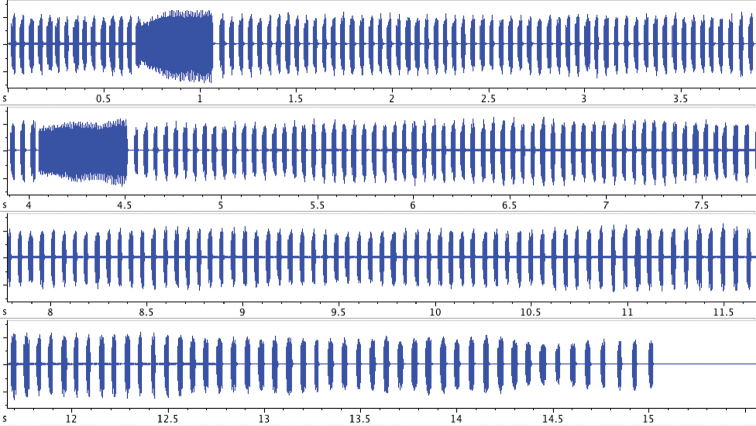
Oscillogram of the song selection of *Cicadivetta goumenissa* at the end of the song with long sequences of SE and with decreasing repetition rate.

## Discussion

Why this cicada species is present only in such a restricted area, as described above, is one of the most puzzling questions concerning *Cicadivetta goumenissa*. We do not see any reason why this cicada would not be able to move to other places, disperse and acquire similar habitats nearby. One possible explanation is that they are, unlike other cicadas, in the larval stage bound to restricted plant species. However, we do not have yet any sound evidence for this hypothesis. Nevertheless, the distribution of endemic cicada species of Evia shows clear similarities with the distribution of endemic plant species on this island (Gogala et al. 2011). Some endemic plants of the Peloponnese are also restricted to small mountainous areas similar to that of the present cicada, as is described by Kit Tan and Iatrou (2006). But we do not know if any of them is in any way connected with this insect.

**Figure 5.**
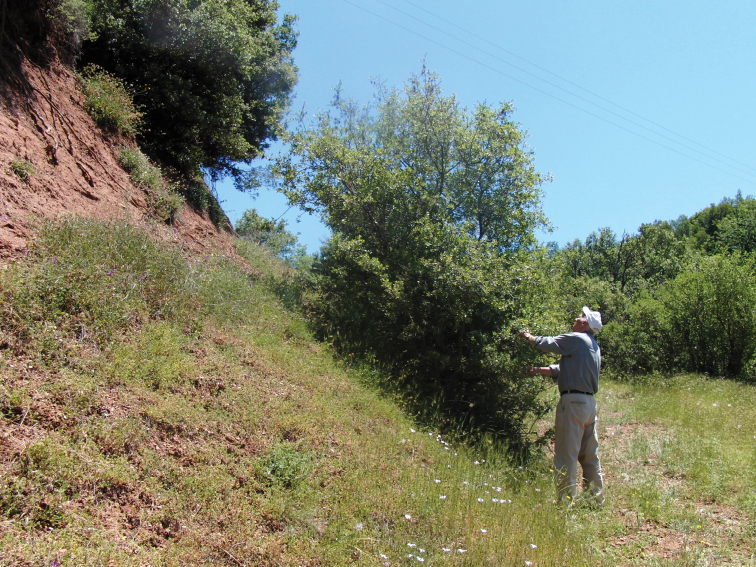
The garrigue or phrygana habitat in the locus typicus of *Cicadivetta goumenissa* near Goumenissa.

The type of vegetation in the distribution area of *Cicadivetta goumenissa* is everywhere very similar and can be described as garrigue or phrygana vegetation with Kermes Oak (*Quercus coccifera*) and other typical shrubs (Fig. 5).

Another possibility would be that this species was in the geological past confined to a small refugium and adapted to particular environmental conditions, which can be found only in the described habitat in this area. We have seen that one environmental parameter, the elevation of localities with *Cicadivetta goumenissa*, is in all cases very similar. The lowest value is according to our measurements 670 m, the highest 860 m and the average 750 m. Is here hidden a hint for its limited distribution?

In the paper with the original description of *Cicadivetta goumenissa* we have already mentioned the unusually high repetition rate of short echemes in the song (13-22 SE/s). In the southern part of Greece there are some other species with a high SE rate such as *Cicadivetta flaveola* (7-11 SE/s), which is found everywhere on the Peloponnese and in some other localities on the Greek mainland. This species occurs also in the distribution area of *Cicadivetta goumenissa* in sympatry with the latter. Another example is also *Cicadivetta carayoni* (Boulard, 1982), an endemic species of Crete with a similar rate of SE in its song (Trilar and Gogala 2010), which according to Puissant and Sueur (2010) also belongs to the same genus. But even many species of the genus *Cicadetta* – e.g. *Cicadetta hannekeae* Gogala, Drosopoulos & Trilar, 2008, *Cicadetta macedonica* Schedl, 1999, *Cicadetta dirfica* Gogala, Trilar & Drosopoulos, 2011 or *Cicadetta kissavi* Gogala, Drosopoulos & Trilar, 2009 - all occurring in various regions of Greece, share with these species long sequences of short echemes and some long echemes. Nevertheless, none of them reaches such a high repetition rate of short echemes in their calling songs, as does *Cicadivetta goumenissa*. Similarly high repetition rates of SE were reported for some western European species like some species of the genus *Tettigettalna* (Puissant and Sueur 2010).

## Conclusions

*Cicadivetta goumenissa* occurs particularly in early June. It is, as expected, not restricted to the neighbourhood of the village Goumenissa, but can be found also in surrounding localities with a similar habitat. Nevertheless, we found it only in an area of about 15 ×25 km. From the new sound recordings here analysed we came to the conclusion that also the phrases with long echemes and without or with only a few short echemes are a regular component of its song and not just an exception.
